# Personalized Neuromodulation: A Novel Strategy for Improving Tinnitus Treatment

**DOI:** 10.3390/jcm12226987

**Published:** 2023-11-08

**Authors:** Seung Yeon Jeon, Jung Ho Choi, Sun Seong Kang, Yong-Hwi An, Hyun Joon Shim

**Affiliations:** Department of Otorhinolaryngology-Head and Neck Surgery, Nowon Eulji Medical Center, Eulji University School of Medicine, Seoul 01830, Republic of Korea; syjeon1993@eulji.ac.kr (S.Y.J.); choi201342@gmail.com (J.H.C.); 20210749@eulji.ac.kr (S.S.K.); raindrop98@eulji.ac.kr (Y.-H.A.)

**Keywords:** neuromodulation, tinnitus, transcranial magnetic stimulation, transcranial direct-current stimulation

## Abstract

This study evaluated the efficacy of personalized neuromodulation, where treatment modalities are chosen based on the patient’s responses in a pilot trial. A total of 71 patients with tinnitus were divided into two groups: a personalized group and a randomized neuromodulation group. In the personalized group (*n* = 35), repetitive transcranial magnetic stimulation (rTMS) and transcranial direct-current stimulation (tDCS) were assessed in a pilot trial, and responsive modalities were administered to 16 patients, while the non-responders (*n* = 19) were randomly assigned to rTMS, tDCS, or combined modalities. Patients in the randomized group (*n* = 36) were randomly allocated to rTMS, tDCS, or combined modalities. The Tinnitus Handicap Inventory (THI) score improvement after 10 sessions of each neuromodulation was significantly greater in the personalized group than in the randomized group (*p* = 0.043), with no significant differences in tinnitus loudness, distress, or awareness. The treatment success rate was highest in the personalized responder subgroup (92.3%), and significantly greater than that in the non-responder subgroup (53.0%; *p* = 0.042) and the randomized group (56.7%; *p* = 0.033). Personalized neuromodulation, where the treatment modality is chosen based on the patient’s responses in a pilot trial, is an advantageous strategy for treating tinnitus.

## 1. Introduction

Tinnitus is the conscious awareness of a tonal or composite noise for which there is no identifiable corresponding external sound source. Tinnitus disorder is defined when it is associated with emotional and/or cognitive dysfunction, as well as autonomic arousal, leading to behavioral changes and functional disability [1]. The current evidence suggests that the causes of tinnitus are multifactorial, and such heterogeneity makes it difficult to understand the mechanisms of tinnitus development [2]. One classical theory suggests that it may result from maladaptive plastic changes in the brain as a result of hearing impairment. Auditory deafferentation caused by hearing loss leads to the functional coupling of auditory and non-auditory areas, resulting in the reorganization of the tonotopic map [3]. Neurobiological and neuroimaging studies have shown that abnormal activities in the non-auditory regions associated with cognitive and attentional functions as well as limbic processes contribute to the unpleasant and distressing aspects of tinnitus [2,4,5]. In particular, the dorsolateral prefrontal cortex (DLPFC) and auditory cortex (AC) are associated with tinnitus. The DLPFC is involved in auditory processing and perception, auditory attention, modulation of the input to the primary AC, as well as various cognitive functions [6,7].

Since the early 2000s, studies have investigated potential treatment options for tinnitus that are based on the concept of reducing the pathological hyperactivity of the auditory network [8,9]. Neuromodulation induces neuroplastic changes by interrupting the aberrant neural activity and thus may alter the tinnitus perception. Repetitive transcranial magnetic stimulation (rTMS) uses a brief, focused magnetic pulse that is directed onto the scalp through a stimulation coil to induce a localized electrical field in the brain. These pulses can be delivered repetitively at various frequencies and intensities. Different frequencies of rTMS, such as low (e.g., 1 Hz) and high (e.g., 10 Hz) frequencies, are believed to exert distinct effects on the interconnected brain regions beneath the stimulation coil [10]. Compared with healthy controls, individuals with tinnitus display heightened functional connectivity between the limbic and cortical regions, as well as increased connectivity among areas responsible for auditory and attentional processing. Individuals with tinnitus also exhibit an elevated resting-state functional connectivity, predominantly within the auditory network, indicating amplified hyperactivity [11]. Low-frequency rTMS (≤1 Hz) decreases neural activity within the targeted brain regions, and it has been used to alleviate hyperactivity in the auditory cortex of individuals with tinnitus [12]. A systemic review of rTMS parameters in a tinnitus trial showed that a lower rTMS stimulation intensity and lower number of pulses were associated with significant effects in verum rTMS arms [13]. This suggests that when higher stimulation intensities are applied, there is a shift toward excitatory responses, away from inhibitory effects.

Transcranial direct-current stimulation (tDCS) is a noninvasive procedure in which a mild current is applied to modify the polarity of cortical neurons and hence modulate cortical activity. Anodal tDCS increases and cathodal tDCS decreases the excitability of the underlying cortex. Anodal tDCS targeting the left temporoparietal region is hypothesized to mitigate tinnitus symptoms by influencing the cortical and subcortical regions, including the Brodmann region and components of the limbic system [9,14].

Numerous studies have sought to increase the effectiveness of interventions to treat tinnitus. However, the heterogeneity of tinnitus results in highly variable individual responses to a given treatment, even from one trial to another when the same treatment is employed. From this perspective, there is a strong rationale for pursuing the personalization of tinnitus treatment [15,16]. In a similar context, recent studies have made attempts at personalized neuromodulation. In the preliminary stage, the feasibility of personalization in rTMS was explored via experimentation. Various protocols involving stimulation frequency, stimulation position, and the laterality of brain stimulation were tested on two different days, effectively demonstrating the consistency of parameters required to achieve the best tinnitus suppression for each individual. These findings underscore the potential for developing individualized rTMS protocols [17,18,19].

Personalization was achieved via pilot tests involving distinct rTMS protocols (1, 10, or 20 Hz, each consisting of 200 pulses) administered over both the left and right temporal cortex. Patients were then randomly assigned to either personalized rTMS (comprising 2000 pulses using the responsive protocol) or standard rTMS (1 Hz; 2000 pulses over the left temporal cortex). The personalized protocol had a greater effect than random stimulation [19]. An analogous method used a similar protocol, where frequencies of 1, 5, 10, and 20 Hz and continuous theta burst stimulation were consecutively administered to the left and right temporoparietal lobes and DLPFC during the experimental session. The responders underwent nine sessions of a composite protocol integrating the most efficacious frontal and temporoparietal stimulation protocols, whereas the non-responders underwent a conventional protocol (20 Hz over the left DLPFC and 1 Hz over the left temporoparietal cortex). There was a numerically, but not significantly, greater reduction in tinnitus in the personalized group compared with the standardized protocol group [20].

One study compared the treatment outcomes between rTMS and tDCS and revealed comparable results in reducing tinnitus in terms of the changes in Tinnitus Handicap Inventory (THI) scores, Numeric Rating Scale (NRS) scores, and duration [21]. Further studies suggest that combining neuromodulation techniques (rTMS + tDCS) improves the treatment outcomes. A single session of tDCS combined with rTMS significantly improved the intensity of symptoms of tinnitus and distress compared with rTMS or tDCS alone [22]. However, there is no established consensus about which neuromodulation modality is most effective for relieving tinnitus. Furthermore, there are no established methods for selecting specific modalities for individual patients with tinnitus or protocols for applying each modality.

There have been studies focused on finding personalized rTMS protocols [17,18,19,20], but there has been no research pursuing which neuromodulation modality, either rTMS or tDCS, to choose for individual tinnitus patients. We hypothesized that applying a neuromodulation modality that elicited a positive response in a pilot trial could achieve better treatment outcomes than randomly selecting a treatment modality.

To verify this hypothesis, we compared the treatment effects and frequency of side effects between patients who received personalized treatment, namely, treatment with a neuromodulation modality selected based on the responses observed during a pilot trial (personalized group), and patients who were randomly allocated a neuromodulation modality (randomized group). Patients in the personalized group without response to any of the neuromodulation modalities in the pilot trial were randomly allocated a treatment modality to ensure a fair comparison with the randomized treatment group. This study sought to evaluate the potential improvements achieved by personalized neuromodulation, where the choice of neuromodulation modality is determined based on the patient’s responses in a pilot trial.

## 2. Materials and Methods

### 2.1. Patients

This study enrolled patients who met the following four criteria: (1) duration of tinnitus exceeding 3 months, (2) aged 18 years or older but under 80 years, (3) complaints of tinnitus-related discomfort, and (4) resistance to pharmacotherapy or tinnitus-retraining therapy. Patients with any of the following were excluded: (1) diagnosis of other otologic diseases, such as otitis media, acoustic tumor, or Meniere’s disease; (2) tinnitus accompanied by sudden hearing loss; (3) suspected somatic tinnitus with complaints of temporomandibular joint and cervical pain; (4) objective tinnitus, including pulsatile tinnitus; (5) history of epilepsy, an implanted heart pacemaker, or an implanted cardioverter-defibrillator; and (6) pregnant women. Patients were not allowed to concurrently participate in other research studies or pursue other treatments for tinnitus during their involvement in this study. A total of 71 patients were enrolled between April 2022 and July 2023. Out of the 71 patients, 35 were assigned to the personalized group, and 36 were assigned to the randomized group in a random manner. This clinical trial was approved by the Institutional Review Board (IRB) of Nowon Eulji Medical Center (IRB no. EMCIRB 18-69) and was conducted in accordance with the revised Helsinki Declaration of 2000. On the first day of the trial, all patients provided written informed consent.

### 2.2. Study Procedures

During the initial visit, patients in the personalized group underwent rTMS and tDCS as pilot trials in a consecutive and random order to establish an individualized protocol for each patient. If patients in the pilot phase observed a reduction of more than 1 point in at least one of two questionnaires for tinnitus loudness and distress, the responsive modality (rTMS, tDCS, or combined) was subsequently applied for the following 10 sessions over a 2-week period. We referred to this group as the ‘personalized responder subgroup’. Patients who showed no response in the pilot trial were randomly assigned to one of the three neuromodulation modalities for the subsequent 10 sessions over a 2-week period. We referred to this group as the ‘personalized non-responder subgroup’.

**Figure 1:**
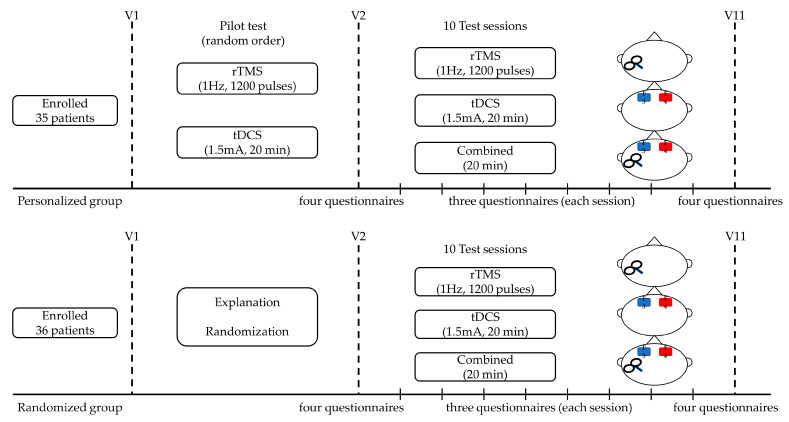
Outline of the study design. During visit 1 (V1), patients in the personalized group underwent a pilot test to determine the modality, whereas patients in the randomized group were informed about the clinical study and randomly assigned to rTMS, tDCS, or combined modalities without a pilot trial. Both groups underdwent the appropriate neuromodulation intervention for 10 sessions over 2 weeks, on five consecutive days per week. Four questionnaires: THI, Numeric Rating Scale for tinnitus loudness, distress, and awareness; three questionnaires: Numeric Rating Scale for tinnitus loudness, distress, and awareness.

Patients in the randomized group were informed about the clinical study during the initial visit and were allocated randomly, using a randomized number list, to receive rTMS, tDCS, or combined modalities without undergoing a pilot trial. These patients received their assigned neuromodulation for 10 sessions over a 2-week period (Figure 1).

To assess tinnitus symptoms subjectively, the patients completed four questionnaires: (1) NRS for tinnitus loudness (ranging from 0 = not at all loud to 10 = extremely loud); (2) NRS for tinnitus distress (ranging from 0 = not at all annoying to 10 = extremely annoying); (3) NRS for tinnitus awareness (patients indicated the percentage of times they were aware of tinnitus over a day, at intervals of 10%); and (4) the THI [23,24]. The changes in tinnitus intensity and distress were assessed immediately after the pilot trial to determine the neuromodulation modality in the personalized group. For all patients in both groups, the tinnitus intensity, distress, and awareness questionnaires were completed before each neuromodulation session and after the 10th session. The THI was completed before the 1st session and after the 10th session. We calculated the post-treatment changes in tinnitus loudness, distress, awareness, and THI by subtracting the score after the 10th session from the score before the 1st session. Treatment success was defined as a reduction of more than 1 point on the 10-point scale for tinnitus intensity or distress, a decrease of more than 10% in the awareness score, or a reduction of more than 10% in the THI score.

### 2.3. Neuromodulation

rTMS was administered using a magnetic stimulation therapy system (ALTMS, REMED, Seongnam, Republic of Korea) with a figure-of-eight coil (field strength ~2.5 Tesla). Prior to each rTMS session, the resting motor threshold was determined by stimulating the motor cortex and measuring the minimum stimulation energy required to elicit five consecutive twitches of the right pollicis brevis muscle. Stimulation was subsequently administered at 100% of the resting motor threshold, at a frequency of 1 Hz, for 1200 pulses (20 min) per session. Ten sessions were conducted over 2 weeks, on five consecutive days each week. The coil was positioned between T3 and P3 using the 10–20 international EEG system for stimulation based on previous reports showing that stimulation laterality does not impact the results and that stimulation of the left temporoparietal cortex is effective irrespective of tinnitus laterality [25,26].

For tDCS, direct-current stimulation of 1.5 mA was applied to both frontal areas for a period of 20 min using a transcranial electrical stimulator (DC-STIMULATOR PLUS, neuroConn GmbH, Ilmenau, Germany). The anode was placed on the left frontal area (F3), and the cathode was placed on the right frontal area (F4) [9,22].

For the combined modalities, tDCS and rTMS were simultaneously administered for 20 min using the protocols described above.

### 2.4. Statistical Analyses

IBM SPSS Statistics (version 29.0, 2023) was used for statistical analyses. Descriptive values are shown as means (standard deviation) unless otherwise stated. The score changes in each questionnaire were calculated by subtracting the post-assessment scores from the pre-assessment scores. Comparisons between the personalized and randomized groups were made using independent *t*-tests for continuous variables, or χ^2^ tests or Fisher’s exact tests for categorical variables. Treatment effects were assessed in three groups (a personalized responder subgroup, personalized non-responder subgroup, and randomized group) and in each modality (rTMS, tDCS, and combined) using analysis of variance for THI and the Kruskal–Wallis test for the tinnitus loudness, distress, and awareness scores. Statistical significance was defined as a *p*-value of less than 0.05. To account for multiple comparisons, we adjusted the *p*-value using the Bonferroni correction (*p* < 0.017, where α = 0.05/3).

## 3. Results

### 3.1. Group Allocation and Side Effects

**Figure 2:**
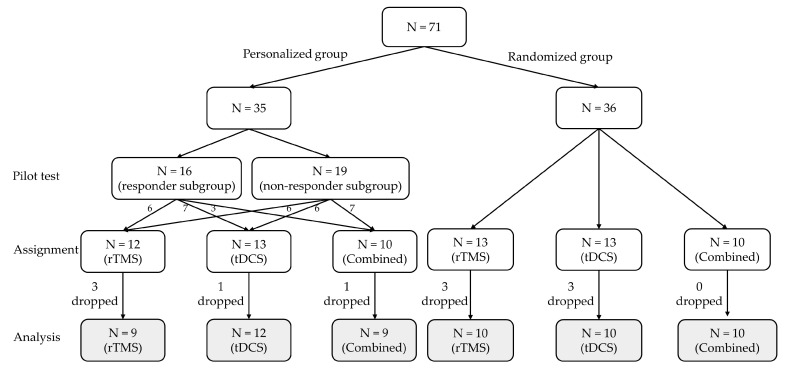
CONSORT flow diagram. In the personalized group (*n* = 35), 16 patients who showed improvement in at least one of the four questionnaires after the pilot trial were included in the responder subgroup and received the treatment modality that elicited a response. The other 19 patients with negative responses (non-responder subgroup) were randomly assigned to one of the three neuromodulation modalities. In the randomized group (*n* = 36), patients were randomly allocated to rTMS (*n* = 13), tDCS (*n* = 13), or combined modalities (*n* = 10) without undergoing a pilot trial. rTMS, repetitive transcranial magnetic stimulation; tDCS, transcranial direct-current stimulation; N, number of patients.

In the personalized group (*n* = 35), 16 patients exhibited a response in the pilot trial and were assigned to the responder subgroup, while the remaining 19 patients who showed no response in the pilot trial were assigned to the non-responder subgroup (Figure 2). The patients were allocated to rTMS (*n* = 12), tDCS (*n* = 13), or combined modalities (*n* = 10). In the personalized group, one individual voluntarily discontinued due to increased tinnitus, and two patients were lost to follow-up among those treated with rTMS. Among patients treated with tDCS or combined modalities, one patient each was lost to follow-up.

In the randomized group (*n* = 36), patients were randomly allocated to rTMS (*n* = 13), tDCS (*n* = 13), or combined modalities (*n* = 10) without undergoing a pilot trial. In the randomized group, two patients allocated to rTMS discontinued treatment due to increased tinnitus and dizziness, respectively, and one case was lost to follow-up. Among patients allocated to tDCS, one discontinued treatment due to escalated tinnitus, one encountered a skin burn, resulting in treatment cessation, and one was lost to follow-up. There were no dropouts among patients allocated to combined modalities (Figure 2). Overall, 30 patients in each of the personalized and randomized groups completed 10 sessions of neuromodulation, and their treatment effects were analyzed. There were no significant differences in the presence of side effects between the personalized and randomized groups (*p* > 0.05).

### 3.2. Patient Characteristics

The mean age of patients in the personalized group was 52.8 years (SD = 17.3; *n* = 35) and 59.4 years (SD = 11.6; *n* = 36) in the randomized group. The mean duration of tinnitus was 24.4 months (SD = 35.3) in the personalized group (*n* = 30) and 24.1 months (SD = 32.6) in the randomized group (*n* = 30). The THI scores before treatment were 51.3 (SD = 22.3) for the personalized group and 40.2 (SD = 23.3) for the randomized group. There were no significant differences in patient demographics, hearing threshold, or pre-treatment questionnaire scores between the personalized and randomized groups (*p* > 0.05; Table 1).

### 3.3. Effects of the Treatments in Each Group

Table 2 compares the questionnaire scores before and after the 10th treatment session in each group. In the personalized group, all scores, except for awareness, improved significantly after treatment (*p* < 0.05 for all). After dividing this group into responders and non-responders, there were significant improvements in all scores, except for awareness, in the responder subgroup (*p* < 0.05 for all). In comparison, there were no significant improvements in any of the scores in the non-responder subgroup (*p* > 0.05 for all). In the randomized group, there were significant improvements in the loudness and distress scores (*p* < 0.05 all).

### 3.4. Comparisons of Changes in the Questionnaire Scores between Groups

**Figure 3:**
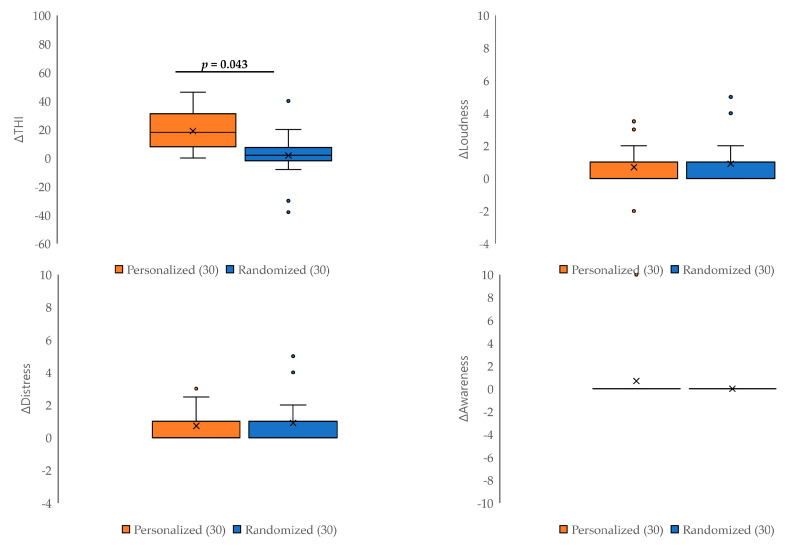
Comparison of changes in the questionnaire scores between the personalized and randomized groups. The improvement in the THI score was significantly greater in the personalized group than in the randomized group (*p* = 0.043). There were no significant differences in the changes in tinnitus loudness, distress, or awareness scores between the two groups (*p* > 0.05 for all).

Figure 3 shows the pre- and post-treatment differences in tinnitus loudness, distress, awareness, and THI in the personalized and randomized groups. A significantly greater improvement in THI score was observed in the personalized group compared with the randomized group (*p* = 0.043). However, there were no differences between the two groups for the changes in tinnitus loudness, distress, and awareness (*p* > 0.05 for all).

**Figure 4:**
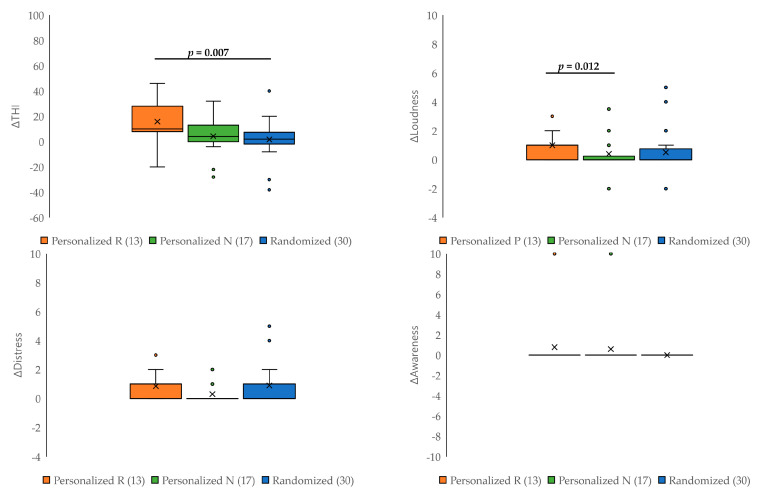
Comparison of changes in questionnaire scores between the personalized responder subgroup (Personalized R), the personalized non-responder subgroup (Personalized N), and the randomized group. The improvement in THI score was greatest in the responder subgroup and was significantly greater than that in the randomized group (*p* = 0.007). A significant improvement in the tinnitus loudness score was observed in the responder subgroup compared with the non-responder subgroup (*p*_Bonf_ = 0.012).

The personalized group was further divided into responders and non-responders to the pilot test, and the changes in the four questionnaire scores were compared between the responder subgroup, the non-responder subgroup, and the randomized group (Figure 4). The improvement in the THI score was greatest in the responder subgroup, with a significant difference compared with the randomized group (*p* = 0.007; Figure 4). Significant improvements in tinnitus loudness were also observed in the responder subgroup compared with the non-responder subgroup (*p*_Bonf_ = 0.012). However, no statistically significant differences in tinnitus distress and awareness were detected across the three groups (*p*_Bonf_ > 0.017 for all).

### 3.5. Comparison of the Treatment Success Rates between Groups

**Figure 5:**
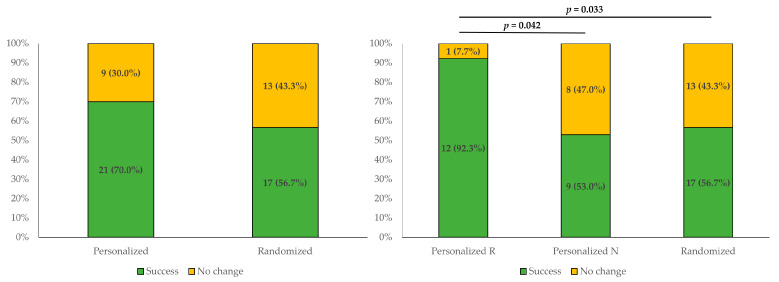
**Comparison of treatment success rates among the study groups**. The treatment success rates for the personalized group and the randomized group were 21/30 (70.0%) and 17/30 (56.7%), respectively, which were not significantly different (*p* > 0.05). When the personalized group was divided based on their response to the pilot trial, the success rate was 12/13 (92.3%) for the responder subgroup (Personalized R), 9/17 (53.0%) for the non-responder subgroup (Personalized N), and 17/30 (56.7%) for the randomized group. The response rate was significantly greater in the responder subgroup than in the non-responder subgroup (*p* = 0.042) and the randomized group (*p* = 0.033).

Figure 5 shows the percentage of patients that showed responses after 10 sessions of treatment in each group in terms of changes in tinnitus loudness, distress, and awareness, or THI scores. “Treatment success” was defined as a decrease of more than 1 point on the 10-point scale for tinnitus intensity or distress, a decrease of more than 10 points in the awareness score, or a reduction of more than 10% in the THI score. The treatment success rates for the personalized group and the randomized group were 21/30 (70.0%) and 17/30 (56.7%), respectively, which were not significantly different (*p* > 0.05). When the personalized group was divided into subgroups based on responses to the pilot test, the success rates were 12/13 (92.3%), 9/17 (53.0%), and 17/30 (56.7%) in the responder subgroup, the non-responder subgroup, and the randomized group, respectively. The success rate was significantly greater in the responder subgroup than in the non-responder subgroup (*p* = 0.042) and the randomized group (*p* = 0.033).

### 3.6. Comparisons between Neuromodulation Modalities

**Figure 6:**
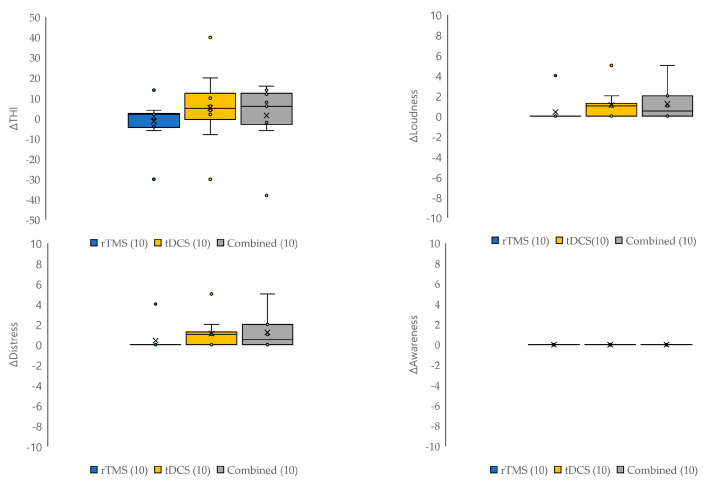
Comparisons of changes in questionnaire scores according to the treatment modalities. The effects of each neuromodulation modality (rTMS, tDCS, and combined modalities) were analyzed in the randomized group. There were no statistically significant differences in the changes in tinnitus loudness, distress, awareness, and THI scores between the three treatment modalities (*p* > 0.05 and *p*_Bonf_ > 0.017 for all).

The differences in treatment effects and the frequency of side effects for each neuromodulation modality were only analyzed in the randomized group. There were no statistically significant differences between the three modalities (rTMS, tDCS, and combined modalities) in the changes in tinnitus loudness, distress, awareness, THI scores (Figure 6), and the frequency of side effects (*p* > 0.05 and *p*_Bonf_ > 0.017 for all).

## 4. Discussion

The success rates of tinnitus patients with rTMS, based on THI scores, ranged from 46% to 51% in prior studies [27]. Additionally, the duration of tinnitus is recognized as an important factor that influences the overall treatment [28]. Among various protocols tested to treat tinnitus, rTMS targeting the temporoparietal junction midway between T3 and P3 shows the greatest efficacy [8,29]. Various tDCS protocols have also been used to optimize the stimulation site, including daily single sessions lasting from 15 to 20 min with current intensities ranging from 1 to 2 mA. The bifrontal DLPFC approach achieved favorable outcomes in patients with tinnitus comorbid with depression and anxiety [30]. Among patients with tinnitus treated with tDCS, the cumulative success rates ranged from 35% to 42% in prior reports [31,32,33]. The efficacy of tDCS was augmented by combining it with other treatment modalities, such as hearing aid sound therapy and tailor-made notched music training [34,35]. In our study, after completing 10 sessions, 63.3% of the patients exhibited a response to the neuromodulation intervention. We observed two instances of worsening tinnitus and one case of dizziness among twenty-five patients who underwent rTMS, as well as one occurrence of worsening tinnitus, one case of dizziness, and one case of skin burn among twenty-six patients who underwent tDCS. Common adverse events associated with rTMS include headache and localized pain around the stimulation site [36], whereas individuals who undergo tDCS often report mild tingling or itching sensations beneath the stimulus electrode, and there have been documented cases of persistent skin lesions due to skin burns, particularly in the frontal scalp region [37].

Previous studies have extensively investigated the concept of personalizing neuromodulation [17,18,19,20]. The outcomes of such investigations have demonstrated both favorable and non-significant results using personalized approaches. One study reported a notable improvement in tinnitus questionnaire scores when a responsive treatment was administered, showcasing an improvement compared with the standard treatment [20]. In contrast, another study showed a numerical but not statistically significant advantage of personalized treatment in terms of the THI, with the discrepancy potentially attributed to the small sample size because only six patients received personalized treatment in that study [19]. In this study, the response rate for the pilot trial of rTMS based on tinnitus loudness or distress was 25.7% (9/35). In other studies, the response rate for pilot trials of rTMS based on superior tinnitus loudness control compared with sham stimulation ranged from 27% to 55% [18,19,20].

This study is the first attempt to investigate the advantages of selecting a treatment modality based on the patient’s responses to a pilot trial. In this study, the personalized group exhibited a significant improvement in their THI scores compared with the randomized group. However, there were no substantial differences in tinnitus loudness, distress, and awareness scores between the groups. This may be attributed to the nature of the THI, which, unlike other questionnaires focused solely on tinnitus distress, loudness, and awareness, encompasses 25 questions spanning functional, emotional, and catastrophic subscales, with a maximum cumulative score of 100. Consequently, the THI captures a wider range of patient responses, not only assessing the direct impact of tinnitus but also encompassing aspects of overall well-being, thereby providing sufficient granularity to establish statistical distinctions [24]. Indeed, neuromodulation also influences quality of life and affection in addition to its effects on tinnitus [38,39]. Furthermore, treatment outcomes were not influenced by hearing status when categorizing patients into normal hearing and hearing disturbance groups based on a hearing level above 25 dB in one or both ears. In both the personalized and randomized groups, there were no significant differences in the changes in tinnitus loudness, distress, awareness, and THI scores between the normal and hearing disturbance groups.

In a previous study, combined modalities yielded a greater therapeutic effect than individual treatments [22]; however, in this study, we did not find a superior effect of the combined modalities compared with the single modalities. In contrast with our study, which used simultaneous modalities, in the previous study, stimulation was administered consecutively. The differences in stimulation duration could potentially influence the treatment outcomes. Concurrent application of rTMS and tDCS enhanced motor-evoked potentials, with a greater effect than either modality used alone [40]. Therefore, further research is warranted to ascertain the superiority of the combined modalities.

This study is the first to validate whether personalizing the treatment modality based on the results of a pilot trial offers advantages over randomly selecting the treatment. An adequate number of patients and results from 10 treatment sessions were used to ensure the findings provide robust support for personalized neuromodulation based on responses to a pilot trial and demonstrate that it is an advantageous strategy for the treatment of tinnitus. The treatment modalities used herein, that is, rTMS, tDCS, and the combined modalities, are widely used neuromodulation techniques. A limitation of this study is the absence of a placebo control group or a sham protocol for neuromodulation. Additionally, the effects observed in the pilot trial were not subjected to reliability testing, including test–retest reliability. Nonetheless, this does not prevent the achievement of the objective of this study, which was to validate the difference in treatment efficacy between personalized treatment and randomized treatment. Furthermore, this study was confined to a single institution, which may introduce selection bias. Lastly, although this study involved a total of 71 patients, the division into different neuromodulation modalities in both the personalized and randomized groups resulted in relatively small sample sizes for each group, typically ranging from 9 to 12 patients. In future studies, a meticulously designed randomized controlled trial should be performed to further optimize the efficacy of neuromodulation.

## 5. Conclusions

This study revealed the potential benefits of a personalized strategy to determine neuromodulation modalities based on responses to a pilot trial for the treatment of tinnitus.

## Figures and Tables

**Table 1:** Demographic characteristics of the enrolled patients.

	Personalized (*n* = 30)	Randomized (*n* = 30)	*p*-Value
**Demographic variables**			
Age	52.8 (17.3)	59.4 (11.8)	0.09
Sex: number of males/females	11/19	14/16	0.60
**Pure tone average**			
Right	19.0 (14.8)	25.9 (23.8)	0.18
Left	26.9 (25.6)	27.0 (24.2)	0.99
<25 dB	*n* = 24/19 (R/L)	*n* = 16/18 (R/L)	
25–40 dB	*n* = 4/7 (R/L)	*n* = 10/8 (R/L)	
40–55 dB	*n* = 1/2 (R/L)	*n* = 2/2 (R/L)	
55–70 dB	*n* = 0/0 (R/L)	*n* = 0/0 (R/L)	
70–90 dB	*n* = 1/0 (R/L)	*n* = 1/1 (R/L)	
>90 dB	*n* = 0/2 (R/L)	*n* = 1/1 (R/L)	
**Tinnitus characteristics**			
Tinnitus duration (months)	24.4 (35.3)	24.1 (32.6)	0.97
Matched loudness (dB SL)	6.4 (4.5)	6.4 (4.1)	0.98
Matched frequency (kHz)	4.5 (3.2)	5.1 (2.7)	0.40
**Questionnaire scores**			
THI scores	51.3 (22.3)	40.2 (23.3)	0.06
NRS loudness	6.1 (2.4)	6.5 (1.8)	0.43
NRS distress	6.2 (2.4)	6.5 (1.7)	0.54
NRS awareness	94.3 (11.9)	97.7 (7.3)	0.19

The data represent the means (with the standard deviation in parentheses). Pure tone average: average of 0.5, 1, 2, and 3 kHz; NRS, Numeric Rating Scale.

**Table 2:** Comparison of the four questionnaire scores before and after treatment in each group.

Group	THI	Loudness	Distress	Awareness
	*p*-Value	*p*-Value	*p*-Value	*p*-Value
Personalized group	0.002 *	0.008 *	0.001 *	0.161
Responder subgroup	0.001 *	0.005 *	0.005 *	0.337
Non-responder subgroup	0.239	0.382	0.056	0.332
Randomized group	0.530	0.002 *	0.002 *	-
Total N	60	60	60	60

* *p* < 0.01.

## Data Availability

The original contributions presented in this study are included in this article. Further inquiries can be directed to the corresponding author.
